# Spectral Flux-Based Convolutional Neural Network Architecture for Speech Source Localization and Its Real-Time Implementation

**DOI:** 10.1109/access.2020.3033533

**Published:** 2020-10-26

**Authors:** YIYA HAO, ABDULLAH KÜÇÜK, ANSHUMAN GANGULY, ISSA M. S. PANAHI

**Affiliations:** Department of Electrical and Computer Engineering, The University of Texas at Dallas, Richardson, TX 75080, USA

**Keywords:** Speech source localization (SSL), direction of arrival (DOA), convolutional neural networks (CNN), beamforming (BF), real-time implementation, hearing improvement (HI)

## Abstract

In this article, we present a real-time convolutional neural network (CNN)-based Speech source localization (SSL) algorithm that is robust to realistic background acoustic conditions (noise and reverberation). We have implemented and tested the proposed method on a prototype (Raspberry Pi) for real-time operation. We have used the combination of the imaginary-real coefficients of the short-time Fourier transform (STFT) and Spectral Flux (SF) with delay-and-sum (DAS) beamforming as the input feature. We have trained the CNN model using noisy speech recordings collected from different rooms and inference on an unseen room. We provide quantitative comparison with five other previously published SSL algorithms under several realistic noisy conditions, and show significant improvements by incorporating the Spectral Flux (SF) with beamforming as an additional feature to learn temporal variation in speech spectra. We perform real-time inferencing of our CNN model on the prototyped platform with low latency (21 milliseconds (ms) per frame with a frame length of 30 ms) and high accuracy (i.e. 89.68% under Babble noise condition at 5dB SNR). Lastly, we provide a detailed explanation of real-time implementation and on-device performance (including peak power consumption metrics) that sets this work apart from previously published works. This work has several notable implications for improving the audio-processing algorithms for portable battery-operated Smart loudspeakers and hearing improvement (HI) devices.

## I. INTRODUCTION

Speech source localization (SSL) estimation generates the important direction information that can be used to improve the performance of many audio/speech signal processing methods such as microphone array beamforming [1]–[4], speech enhancement [4], [5], speech/speaker recognition [6], [7], and hearing improvement (HI) devices such as Roger Select [8] and Roger Table Mic [9]. Many commercial products are available to the public which use some types of microphone arrays and some forms of SSL methods aimed at specific applications. Considering all these, however, the robustness, accuracy, and cost-effectiveness of the SSL-based methods remain a challenging issue, especially in noisy environments at low signal to noise ratios (SNRs).

### A. PRIOR WORK

The previous SSL methods and direction of arrival (DOA) estimators include (i) multiple signal classification (MUSIC) [10], (ii) time difference of arrival (TDOA) based approaches such as generalized cross-correlation (GCC) [11], and multi-channel cross-correlation coefficient (MCCC) [12]. These conventional methods often suffer from the high levels of noise, presence of reverberation, and/or the high computational complexity. Since neural networks-based machine learning (ML) classification has been successfully applied in computer vision and speech recognition, many neural network based DOA estimators have been proposed [13]–[15]. Even though these methods show the improvement in estimation accuracy compared to conventional methods, the results are still unsatisfactory. For example, (i) they still show low-accuracy estimation in the low SNR condition, (ii) most of these methods are highly dependent on (overfitted) to the training data and hard to cover other scenarios, (iii) some of the SSL-based methods still stay at utilizing a small number of microphones, such as the use of two microphones in a conventional method in [16], and neural network based method in [17], which bears the 180° ambiguity problem.

### B. PROPOSED METHOD

**FIGURE 1.**
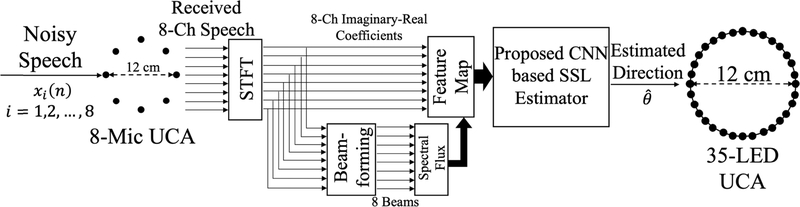
The block diagram of the proposed real-time platform using eight uniform circular array (UCA) of microphones.

In this article, a novel eight-microphone uniform circular array (UCA) based SSL estimator using convolutional neural networks (CNN) is proposed. This work assumes eight participants are sitting around the circular table since it is a common case. Previous CNN based methods such as [17] show that using imaginary-real coefficients as the feature map can work in several realistic environments but still suffer from the background noise especially when SNR is low. As the augmentation of [17], another feature, spectral flux, is included in the feature map. Additionally, a delay-and-sum (DAS) beamformer [18] is added to enhance the SNR before computing spectral flux. Thus, the feature map contains both of the imaginary-real coefficients of the short-time Fourier transform (STFT) and the spectral flux with beamforming which can essentially improve the performance of the proposed estimator. Several microphone array can solve the 180° ambiguity issue such as V-shape, circular (UCA), and spherical arrays. In this work, the UCA of eight microphones is selected for the proposed method. Such structure has been used in many commercial products such as smart loudspeakers [19], [20]. Fig. 1. shows the block diagram of the proposed SSL platform. Noisy speech data is received through the UCA microphones, then the imaginary-real coefficients are calculated by the STFT. Meanwhile, the STFT outputs are sent to a DAS beamforming module (which converts the signals into eight beams), then the spectral flux is generated from the signals of eight beams. The imaginary-real coefficients and the spectral flux are combined and reshaped into the feature map, then fed to the proposed SSL/DOA estimator. Once the direction of the speech source $\hat{\theta }$ is estimated by the algorithm, it will be displayed by turning on the proper LED pointing out the speech source direction. There are 35 LEDs positioned circularly on top of the development board covering the entire 360° azimuth in the horizontal plane. The proposed method has been implemented to run in real-time on the prototyped platform which formed with a Raspberry Pi and an internet-of-things (IoT) development board with UCA microphones. The proposed method has shown excellent performance and accuracy offline or in real-time under realistic noisy environments. The real-time testing was completed in a separate room which is different as the room for the data collection. We selected 8-microphone array because of easy off-the-shelf availability and our developed proprietary software integration with Raspberry Pi over GPIO pins. We selected 8 beams because it was a requirement from our sponsor.

### C. CONTRIBUTIONS

In neural network-based SSL, the feature of imaginary-real coefficients has already been used widely such as the work in [17]. The major contribution of this work is the augmentation of the imaginary-real coefficients with spectral flux plus beamforming. The utilization of spectral flux as one of the features can incorporate temporal dependency between successive signal frames. Since few CNN-based SSL estimators utilize temporal information, we have shown considerable improvement of 8% in accuracy by including spectral flux into the feature set. The beamforming technique essentially improves the performance of the spectral flux-based method. Therefore, a pre-processing stage by beamformer enhances the SNR of the input signal for spectral flux in the proposed method. Typically, CNN models treat each feature vector to be independent of adjacent frames, hence including spectral flux can yield better models that are more aware of voiced-activity-detection (VAD) type activities. Although some models such as recurrent neural network (RNN) can essentially learn the above temporal representations, they are usually more memory intensive and have higher latency than their CNN counterparts. Directly stacking coefficients (imaginary-real or magnitude-phase) from previous frames brings the CNN model temporal awareness, but it requires more expensive computation as compared to spectral flux (due to the longer length of the input feature size). Another contribution of this work is the prototyped platform with beamforming which converts the proposed method from an offline trained model into a real-time SSL estimator. In this article, the proposed SSL estimator only focuses on the eight-class which divides the 360° azimuth into eight directions with 45° resolution, but it does not limit in eight-class. For example, it can be extended to a twelve-class (30° resolution) case by generating a twelve-beams beamformer. The end-products similar like [8] and [9] can be built based on the prototype. The proposed method, therefore, offers both scientific significance and practical importance.

In this article, we use the term “eight-microphone” or “eight-channel” to specify the number of SSL sensors/microphones used. In the figures, we also use the term “MIC” or “CH” denoting the microphone. The term “Beam” denotes the output signal after beamforming.

## II. FEATURE REPRESENTATION FOR TRAINING

The feature representation needs to contain enough information for the estimation purpose. In our proposed method, the imaginary-real coefficients from the STFT and the spectral flux after beamforming are combined as the feature set. The speech information is included in the imaginary-real coefficients of the current frame (i.e. the voiced segments of the speech such as vowels have harmonic characteristics). The spectral flux contains information of the magnitudes for the current frame and the previous frame which provides the model with the short-term memory.

### A. IMAGINARY AND REAL COEFFICIENTS

For the proposed CNN method, the *N*-point STFT is applied to every data frame of the time-domain signal, shown as

(1) $$X_{k}^{i}(m)=\sigma_{k}^{i}(m)+j\tau_{k}^{i}(m)$$

where, $X_{k}^{i}(m)$ stands for the output of *N*-point STFT of $x_{k}^{i}(n)$ (from *i*^*th*^ microphone for *k*^*th*^ frame). $\sigma_{k}^{i}(m)$ denotes the real part of the $X_{k}^{i}(m)$, and $\tau_{k}^{i}(m)$ denotes the imaginary part of $X_{k}^{i}(m)$. *m* denotes the frequency bin. In the proposed method, the real parts $\sigma_{k}^{i}(m)$ and the imaginary parts $\tau_{k}^{i}(m)$ as one of the features feed the CNN models for training, and forming the following vectors,

(2) $$\tau_{k}^{i}={\left[\tau_{k}^{i}(1)\tau_{k}^{i}(2)\ldots \tau_{k}^{i}\left(\frac{N}{2}+1\right)\right]}^{T}$$

(3) $$\sigma_{k}^{i}={\left[\sigma_{k}^{i}(1)\sigma_{k}^{i}(2)\ldots \sigma_{k}^{i}\left(\frac{N}{2}+1\right)\right]}^{T}$$

Using (2) and (3), the feature $\Phi_{k}^{l}$ can be represented by the following matrices,

(4) $$\Phi_{k}^{l}={\left[\tau_{k}^{1}\tau_{k}^{2}\ldots \tau_{k}^{8}\right]}^{T},\;l=1$$

(5) $$\Phi_{k}^{l}={\left[\sigma_{k}^{1}\sigma_{k}^{2}\ldots \sigma_{k}^{8}\right]}^{T},\;l=2$$

where, *l* is the number of feature channel. Hence, $\Phi_{k}^{1}$ represents the imaginary coefficients feature set, and $\Phi_{k}^{2}$ stands for the real coefficients feature set.

### B. SPECTRAL FLUX

The imaginary-real feature can cover the frequency domain information of the speech. However, it only covers *k*^*th*^ signal frame information excluding any relations between adjacent frames. This disadvantage can be resolved by adding spectral flux into the feature set for proposed CNN model which offers the short-time memory. In conventional signal processing SSL methods, the performance of using spectral flux has already been utilized and shown by scholars such as [21]. It is interesting to note that spectral flux works so well without any phase information. The reason could be that instead of the absolute values of the captured samples, spectral flux only contains the relative values (the STFT magnitude difference between successive frames) which are more robust for the disunity issue of microphone array introduced by hardware.

In the proposed method, the signals from eight microphones are converted to the frequency domain by STFT, then processed by the beamforming module. Then they are converted to eight beams. That is,

(6) $$BF_{k}^{q}(\;\text{m})=\frac{1}{L}\sum_{i=0}^{L-1}W^{q,i}(m)X_{k}^{i}(m)$$

(7) $$Y_{k}^{q}(m)=A_{k}^{q}(m)e^{j\theta_{BF}q_{k}^{q}(m)}$$

where, $BF_{k}^{q}(\;m)$ denotes the beamformer output at *q*^*th*^ beam for *k*^*th*^ frame. *L* stands for the total number of the microphones which equals to eight in this work. *W*^*q,i*^(*m*) denotes the finite impulse response (FIR) filter weights in frequency domain at *i*^*th*^ microphone for *q*^*th*^ beam. In (7), $A_{k}^{q}(m)$ is the magnitude of $BF_{k}^{q}(\;m)$, and $\theta_{BF_{k}^{q}(m)}$ stands for the phase of the $BF_{k}^{q}(\;m)$. Hence, the spectral flux coefficients for two successive frames can be calculated as follows,

(8) $$S_{k}^{q}(\;\text{m})=\left|A_{k}^{q}(m)\right|-\left|A_{k-1}^{q}(m)\right|$$

(9) $$S_{k}^{q}={\left[S_{k}^{q}(1)S_{k}^{q}(2)\ldots S_{k}^{q}\left(\frac{N}{2}+1\right)\right]}^{T}$$

where, $S_{k}^{q}(\;\text{m})$ is the magnitude differences between two adjacent frames, and $S_{k}^{q}$ represents the spectral flux for *q*^*th*^ beam and *k*^*th*^ frame. Then the spectral flux-based feature constructed as

(10) $$\Phi_{k}^{l}={\left[S_{k}^{1}S_{k}^{2}\ldots S_{k}^{8}\right]}^{T},\;l=3$$

As (10) shows, spectral flux as the third feature channel has been inserted into feature map $\Phi_{k}^{l}$. The details of the training input formats are discussed in Section III.

## III. CONVOLUTIONAL NEURAL NETWORK MODEL

**FIGURE 2.**
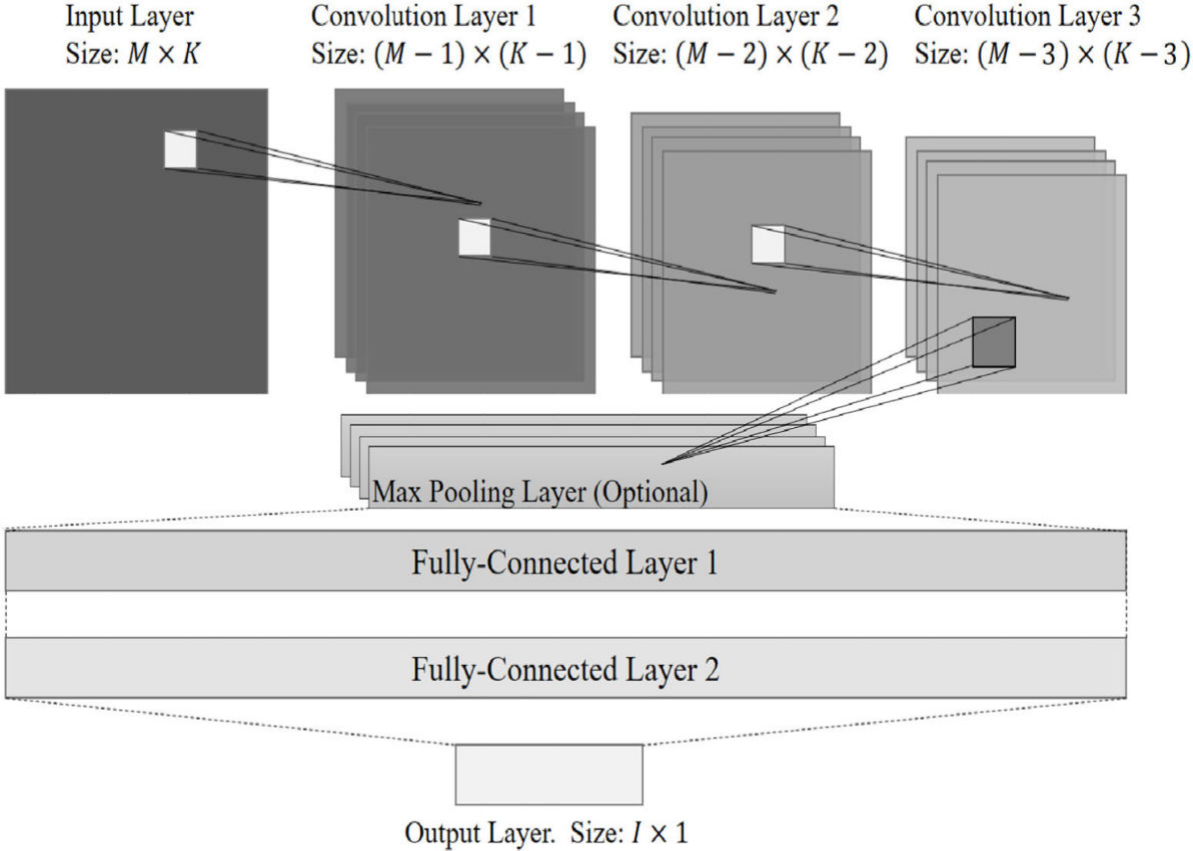
The CNN model of the proposed method. The size of the input layer is 8 × 771. The size of the output layer is 8 × 1.

In this section, the CNN model of the proposed method is presented. The architecture of the proposed CNN model contains one input layer, three convolution layers, one pooling layer, two fully-connected layers, and one output layer. The size of each feature map is *M*×*K*, where *M* = 8, since there are eight microphones/beams, and *K* = (*N*/2+1)×*H*, where *N* is the number of the STFT point. In proposed work, *H* = 3 which stands for the $\Phi_{k}^{1}$, $\Phi_{k}^{2}$ and $\Phi_{k}^{3}$. The CNN model is shown as Fig. 2.

### A. DATA LABELING

In order to train the CNN model, the realistic speech signals have been captured and used to create datasets for training and testing purposes.

The recorded data was labeled and reshaped into the feature set $\Phi_{k}^{l}$. The frame size equals to 30 milliseconds at 16kHz sampling frequency, resulting in 480 samples for each frame. Therefore, the STFT size is set to *N* = 512 points. After STFT, there are (*N*/2+1) × 3 = 771 coefficients, and the total size of the input feature is 8 × 771. The imaginary-real coefficients and spectral flux ($\Phi_{k}^{1}$, $\Phi_{k}^{2}$ and $\Phi_{k}^{3}$) of eight microphones/beams are put into the eight different rows. Each row contains the three features of one microphone or beam pointing at one direction (e.g. the first row contains three features at the direction of 0°). The dataset ${\tilde{\Phi }}_{k}$ can now be denoted as in (11).

(11) $${\tilde{\Phi }}_{k}=\left[\begin{array}{ccc} \tau_{k}^{1T} & \sigma_{k}^{1T} & S_{k}^{1T}\\ \vdots & \vdots & \vdots \\ \tau_{k}^{8T} & \sigma_{k}^{8T} & S_{k}^{8T} \end{array}\right]$$

A ground truth *θ*_*k*_ (for *k*^*th*^ frame) is put at the end of the vector representing the actual direction. It is interesting that rearranging the feature matrix (such as swapping the positions of $\tau_{k}^{iT}$ and $S_{k}^{qT}$) creates an insignificant difference for the final results. It is due to the spatial invariance of the CNN in classification problems [22].

### B. CNN MODEL

Once the pre-processing including labeling and reshaping has been completed, the input feature maps are fed into the CNN model for training. A set of filters of size 2 × 2 in the convolution layer is applied to learn the correlations among all the feature coefficients. Each filter convolves with the first 2 × 2 samples of the input feature map then shifts one step towards the right-hand side to do the next convolution. Each convolutional layer contains 64 filters. After three convolution layers, a pooling layer is followed to downsample the data. The size of the fully-connected layer equals to (*M* − 3) × (*K* − 3) = 5 × 768 = 3840. Then the modeled coefficients are sent to the first fully-connected layer. The rectified linear units (ReLU) activation function [23] is used inside the fully-connected layers. After two fully-connected layers, the coefficients will be mapped to the output layer with the size of *I* × 1, which treats the whole system as a classification problem. In this case, we set the *I* = 8 which means the resolution is 45°. This resolution is used since it can cover typical situations encountered by a user with people around, such as in a business meeting, group conversations, and dining in a restaurant.

The softmax function is applied to generate the probability for each coefficient *θ*_*k*_ inside the output layer. The cross-entropy is used as the lost function. The final SSL - the DOA estimated azimuth angle is then given by,

(12) $${\hat{\theta }}_{k}=arg\;\underset{\theta_{k}}{max}\;p\left(\theta_{k}|\Phi_{k}^{l}\right)$$

where $p\left(\theta_{k}|\Phi_{k}^{1}\right)$ denotes the conditional probability of *θ*_*k*_ using $\Phi_{k}^{1}$, $\Phi_{k}^{2}$ and $\Phi_{k}^{3}$. ${\hat{\theta }}_{k}$ is the final estimated direction (the DOA angle estimate) at *k*^*th*^ frame.

In the experiment setup, the feature sets contain 90 minutes clean speech for each direction with 45° resolution. The CNN models shuffle the feature sets and apply 90 percent of the data to train the model, and 10 percent of the data to validation.

After the whole training is completed, a frozen model is generated as the proposed CNN based SSL estimator. The proposed method has been implemented on the prototyped platform in real-time. Therefore, both of the offline validation/testing results and the real-time performance of the proposed method have been measured. The proposed model is built, trained and implemented based on Tensorflow (version 2.0) [24].

## IV. DATA COLLECTION

The performance of a learning model using a simulating dataset is unconvincing, especially in the realistic scenarios. Therefore, a data collection scheme is presented to obtain a realistic dataset for model training.

### A. DATA COLLECTION SCHEME – THE SETUP

**FIGURE 3.**
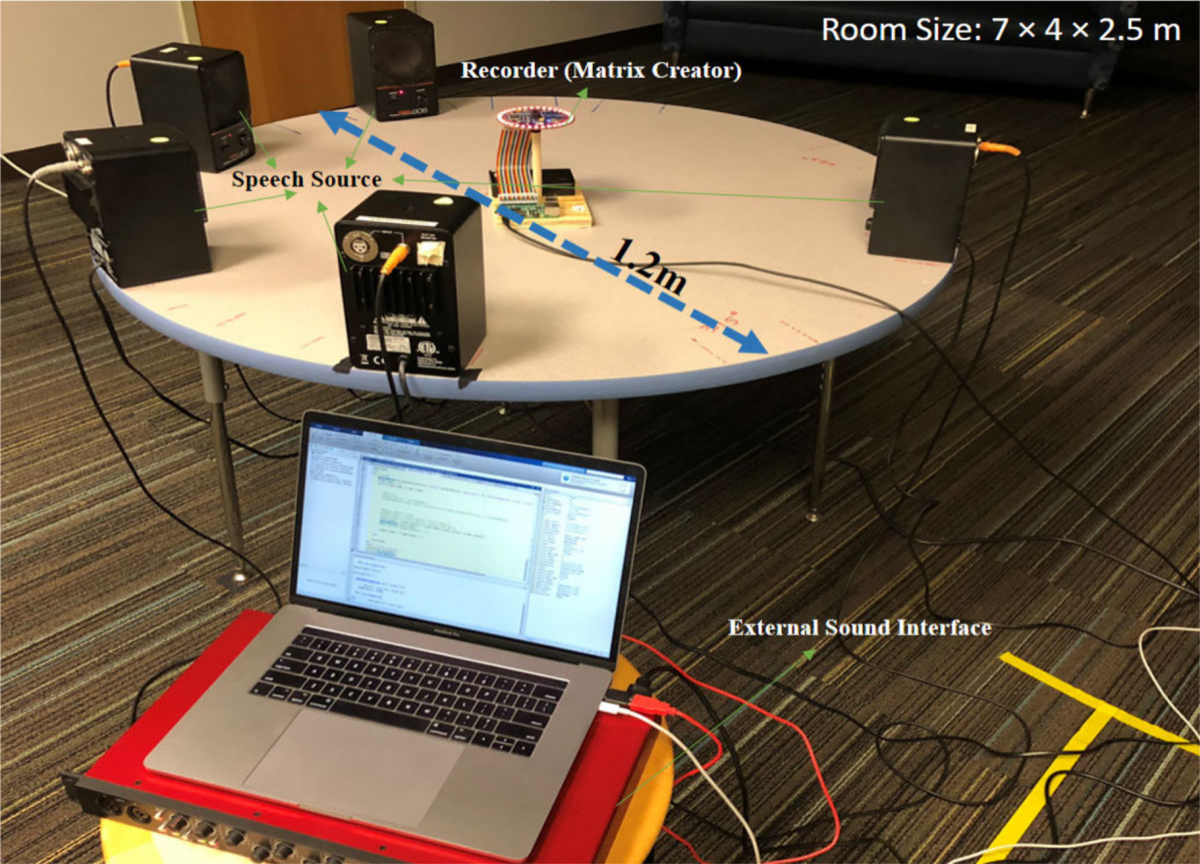
The Setup of Data Collection (Room A).

Fig. 3 shows the setup of the data collection in room A. Multiple loudspeakers are placed at the edge of a circular table. The clean speech signals are played via the loudspeakers while another loudspeaker locating under the table can play the noise creating diffused background noise. All loudspeakers are connected to an external audio interface which is controlled by a script running on a MacBook via a USB3.0 cable. The prototyped platform as the recording device with eight Micro-Electro-Mechanical System (MEMS) microphones sits in the center of the circular table. The training speech is made based on HINT database [25]. The total length of the training speech is 90-minutes long for each direction/loudspeaker. The data collection was completed in room A, B, and C. The real-time testing was completed in room D. The setup information is presented in Table 1. Details of the prototyped platform is presented in section VI.

### B. COLLECTION PROCEDURES

Sound level calibration is required before the collecting session. A sound pressure level (SPL) meter is used to calibrate the output levels of all loudspeakers to 65 dB SPL. The level of the noise loudspeaker is set at different SNRs for conducting the experiments.

After the sound level calibration, the speech signal from the first loudspeaker starts to play while the noise loudspeaker is playing at the same time. The first loudspeaker plays the speech for 90 minutes, then the second loudspeaker starts to play from another location/direction. Using the same manner, the rest of the loudspeakers play speech signals from different directions one after another.

Once the data collection session is done, the recorded audio data will be dissected into different pieces (one single piece stands for one loudspeaker direction). Then the azimuth directions are labeled to corresponding speech pieces as discussed above. The collected dataset is currently available for public use in [26].

## V. MEASURED RESULTS AND DISCUSSION

In this section, we present several offline test results to show the performance of the proposed method (denotes as 8CH-ImagReal-SF-BF) compared with other published methods to the cases we considered. The comparisons are trained/tested with the same dataset as the proposed method. The comparisons include a conventional signal processing SSL estimator based on the generalized cross-correlation (GCC) [27] (denotes as 8CH-GCC), an MLP neural network based eight-microphone SSL estimator using GCC-Phat as the feature set [28] (denotes as 8CH-GCCPhat-MLP), and a CNN-based SSL estimator using the phase of the white noise as the feature set [29] (denotes as 8CH-Phase-WN). We use 8CH-Phase-WN to aim at single speech source localization since the contributions of our proposed work only focus on the single source. Another two comparisons use the same CNN model as the proposed method. One of them uses the feature of the imaginary-real coefficients (same as the published work in [17]) (denotes as 8CH-ImagReal). In order to measure the improvement by beamforming, another method using the imaginary-real coefficients and spectral flux without beamforming is included as well (denotes as 8CH-ImagReal-SF). The experiments include the offline testing and real-time testing. The offline testing is based on the collected data in room A, B, and C. The 10 percent of the collected data is used for testing (90 percent of the collected data is used for training). The real-time experiments were completed in room D with the prototyped platform. The dimension of the rooms is shown in Table 1. The offline measured results are presented in this section, and the real-time test results is presented in section VI.

**FIGURE 4.**
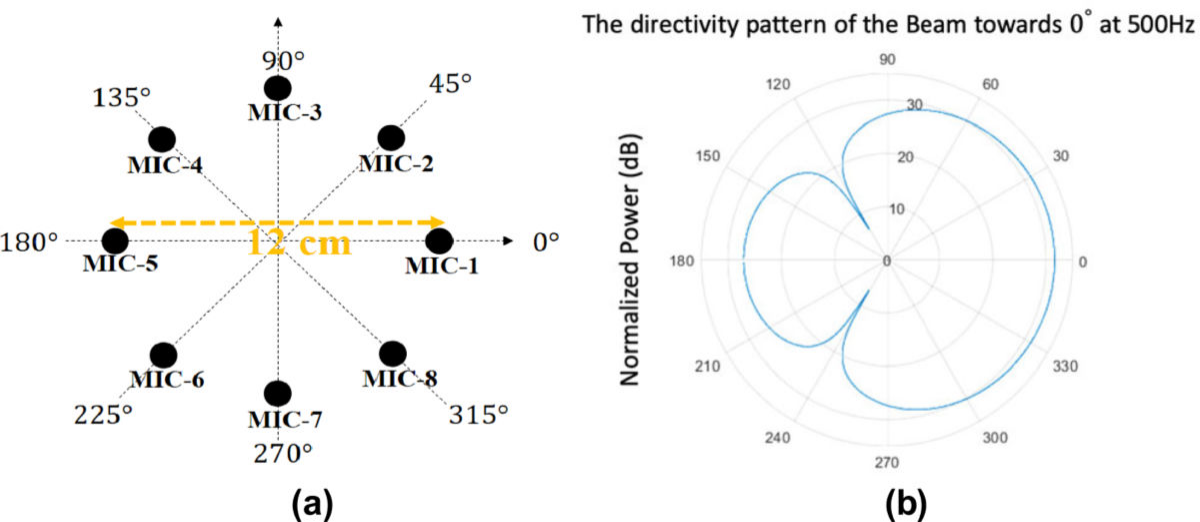
(a) The geometric positions of the eight-microphone UCA, and (b) the directivity pattern of the first beam towards 0° at 500Hz.

Fig. 4. (a) shows the UCA geometric positions. The MIC-1 is located at 0°. DAS beamforming has been used to enhance the SNR for spectral flux feature (DAS modifies the phase information so that phase-related features such as imaginary-real is unsuitable). DAS beamforming has low computation complexity compared to other beamformers such as MVDR [30] which ensures real-time implementation. The directivity pattern of the first beam towards 0° at 500Hz of the beamformer is shown in Fig. 4. (b). Eight linear-phase fractional-delay filters convolve with their corresponding microphone signals to generate the first beam. All eight beams point to their own directions from 0° to 315° and have 45° between every two adjacent beams.

### A. THE PERFORMANCE OF THE PROPOSED METHOD UNDER QUIET CONDITION

In this section, the measured results under quiet and noisy conditions are presented. 90-minutes long collected speech dataset for eight directions are used for training and testing. 90 percent of the collected data is used for training, and the rest is used for the testing. The accuracy is quantified based on the root mean square error. The accuracy (*ACC*) measure is defined by,

(13) $$ACC=\frac{N_{C}}{N_{F}}$$

where, *N*_*F*_ is the total number of the frames per test case and *N*_*C*_ is the total number of the frames with the correct direction estimation. *N*_*C*_ can be denoted as,

(14) $$N_{C}=\sum_{k=1}^{N_{F}}c_{k},\;c_{k}=\{\begin{array}{ll} 0, & \theta_{k}\neq {\hat{\theta }}_{k}\\ 1, & \theta_{k}={\hat{\theta }}_{k} \end{array}$$

where, *c*_*k*_ represents the estimated correction of *k*^*th*^ frame. *θ*_*k*_ is the actual direction and ${\hat{\theta }}_{k}$ is the estimated direction for the *k*^*th*^ frame. *ACC* can present the performance of the estimator partly, because the result is correct only if the estimated direction is same as the actual direction. However, if the estimated direction is only one class different from the actual direction, the *ACC* result will still show the estimation is failed even it just one class different. To quantify the performance additionally, the *ACC*_*w*_ is introduced. It is defined as,

(15) $$ACC_{w}=\frac{{\tilde{N}}_{C}}{N_{F}}$$

where, ${\tilde{N}}_{C}$ denotes the number of the correction frame with a wide angle.

(16) $${\tilde{N}}_{C}=\sum_{k=1}^{N_{F}}{\tilde{c}}_{k},\;{\tilde{c}}_{k}=\{\begin{array}{ll} 0, & \left|\theta_{k}-{\hat{\theta }}_{k}\right|>45^{{}^{\circ}}\\ 1, & \left|\theta_{k}-{\hat{\theta }}_{k}\right|\leq 45^{{}^{\circ}} \end{array}$$

**FIGURE 5.**
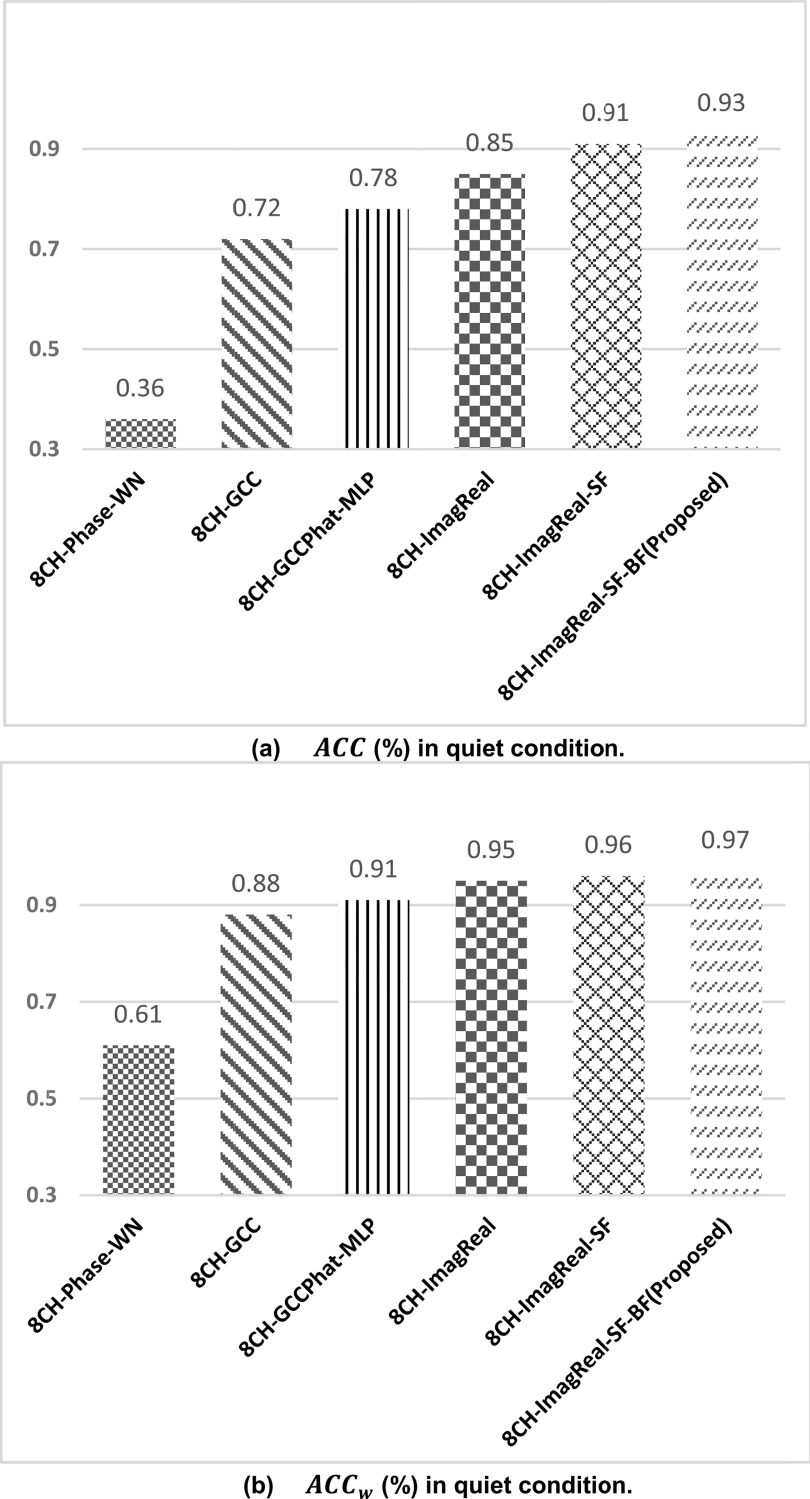
The offline *ACC* results (%) and the *ACC*_*w*_ results (%) of the proposed method and the comparisons under quiet condition. Both of the training and testing used the collected data in Room A, B, C.

**FIGURE 6.**
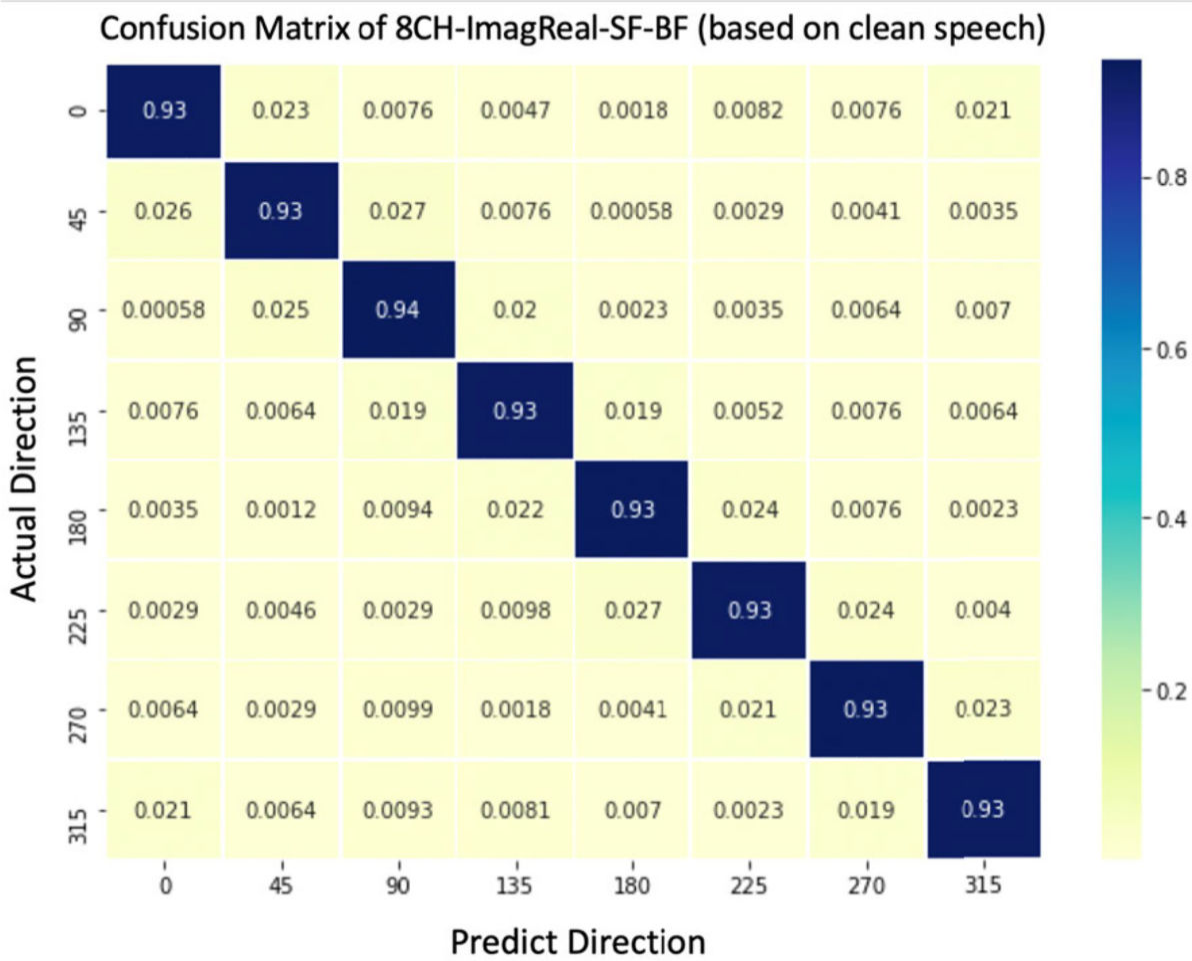
The Confusion Matrix (normalized) of proposed method using clean speech. The training/testing data were collected in Room A, B, C.

In the quiet environment, the *ACC* of the proposed method, 8CH-ImagReal-SF-BF, is measured and compared with other methods including 8CH-GCCPhatMLP, 8CH-Phase-WN, 8CH-GCC, 8CH-ImagReal, and 8CH-ImagReal-SF (Fig. 5(a)). The performance of 8CH-GCC, as a conventional signal-processing based estimator, is worse than most of the other neural network-based estimators except 8CH-Phase-WN. 8CH-Phase-WN did not perform well in our experiments under Quiet conditions. Hence it is removed from our experiments under Noisy condition. The proposed method reaches the best performance with 93% *ACC* among all estimators. The proposed method is better than 8CH-ImagReal which shows the improvement of the combination features (imaginary-real coefficients plus spectral flux) comparing to using imaginary-real coefficients alone. Meanwhile the proposed method is also better than 8CH-ImagReal-SF. This is the improvement by beamforming which boosts the SNR of the input for spectral flux. The confusion matrix of the proposed method shows that the *ACC* results from each direction are stable (Fig. 6). It also shows the proposed method has high *ACC*, meanwhile, the incorrect estimations mostly stay within 45°. The *ACC*_*w*_ results in Fig. 5(b) prove it again by presenting the accuracy with a wider angle. 8CH-ImagReal reaches 95% *ACC*_*w*_ but still lower than the proposed method which reaches the best results again at 97% *ACC*_*w*_. According to both of the *ACC* and *ACC*_*w*_ results, the proposed method is better than the 8CH-ImagReal. This fact proves that for the feature set, the combination of the imaginary-real and the spectral flux with beamforming performs better than using imaginary-real alone.

### B. THE PERFORMANCE OF THE PROPOSED METHOD UNDER NOISY CONDITIONS

**FIGURE 7.**
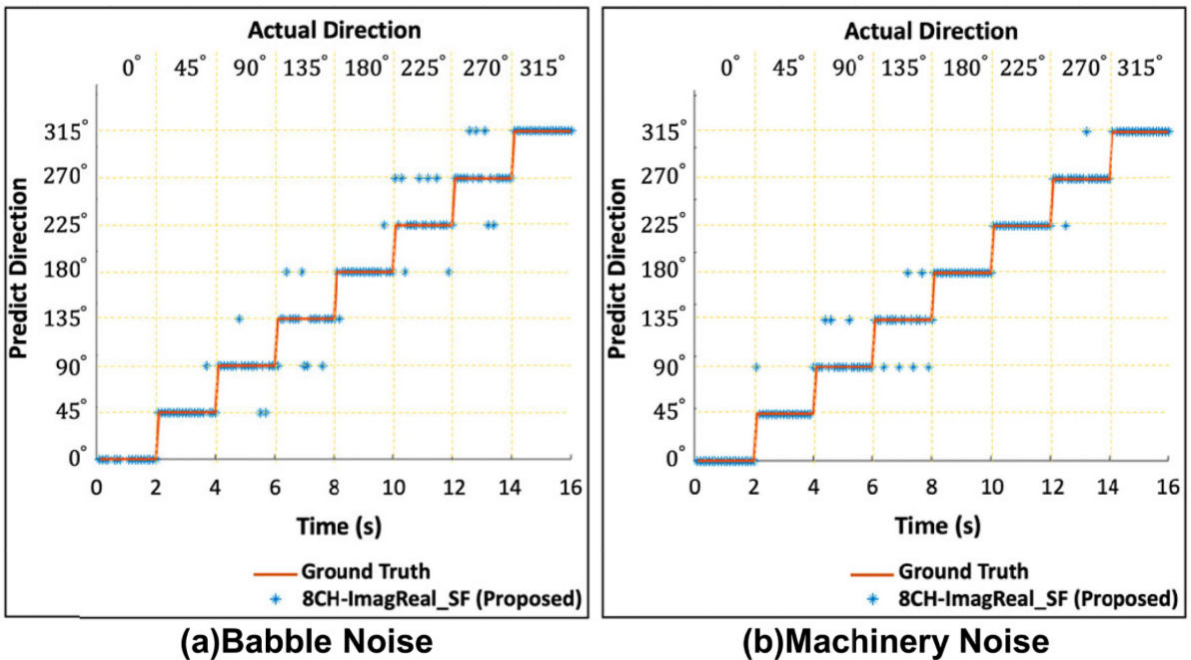
The performance of the proposed method under babble noise or machinery noise at 5dB SNR using 16-second recorded speech.

All the results above are only based on the clean speech signals. In order to test and evaluate the performance of the proposed SSL method, noisy speech data are collected as follows. Speech is played by eight loudspeakers one-by-one circularly placed on a table at 0° to 315° angles with 45° resolution. Meanwhile, noise is played by a loudspeaker placed under the table simulating diffused noise. The setup is presented in Section VI. The proposed method is tested under babble noise or machinery noise both at 5dB SNR. The test speech signal is 16-second long (every two seconds for one direction) and played by each of the eight loudspeakers from each angular direction sequentially. The results are presented in Fig. 7. The x-axis denotes the playing speech in time-domain while a 2-second speech playing from each direction one from another. The y-axis represents the directions. The blue stars stand for the estimated directions, and the ground truth is represented by the red line. The result shows that the *ACC* of the proposed method (at 5dB SNR) is around 90 percent under babble noise (Fig. 7(a)), and the *ACC* even reaches 93% under machinery noise (Fig. 7(b)).

**FIGURE 8.**
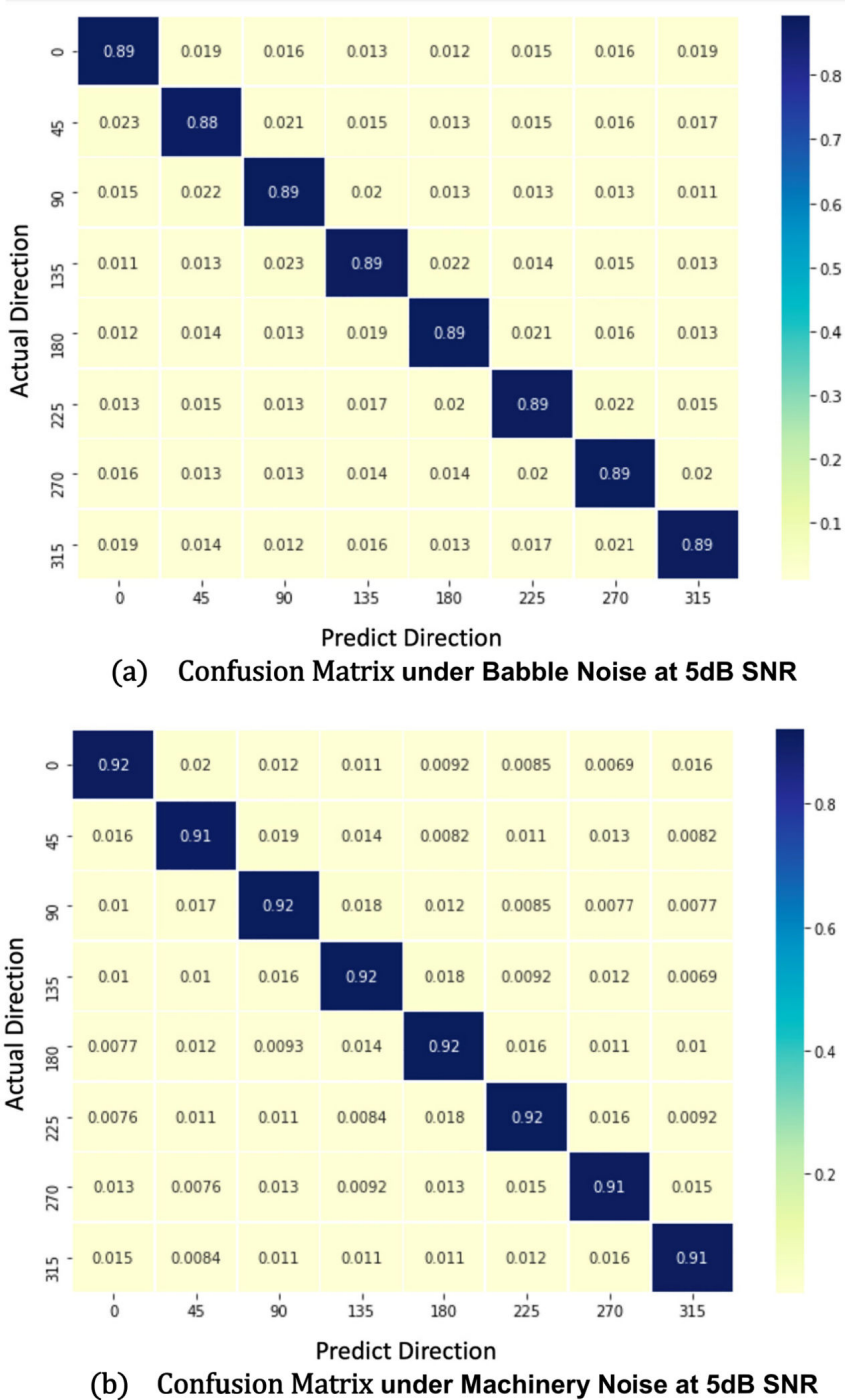
The confusion matrix of the proposed method using speech under (a) babble (b) machinery noise at 5dB SNR. The training/testing data were collected in Room A, B, C.

**FIGURE 9.**
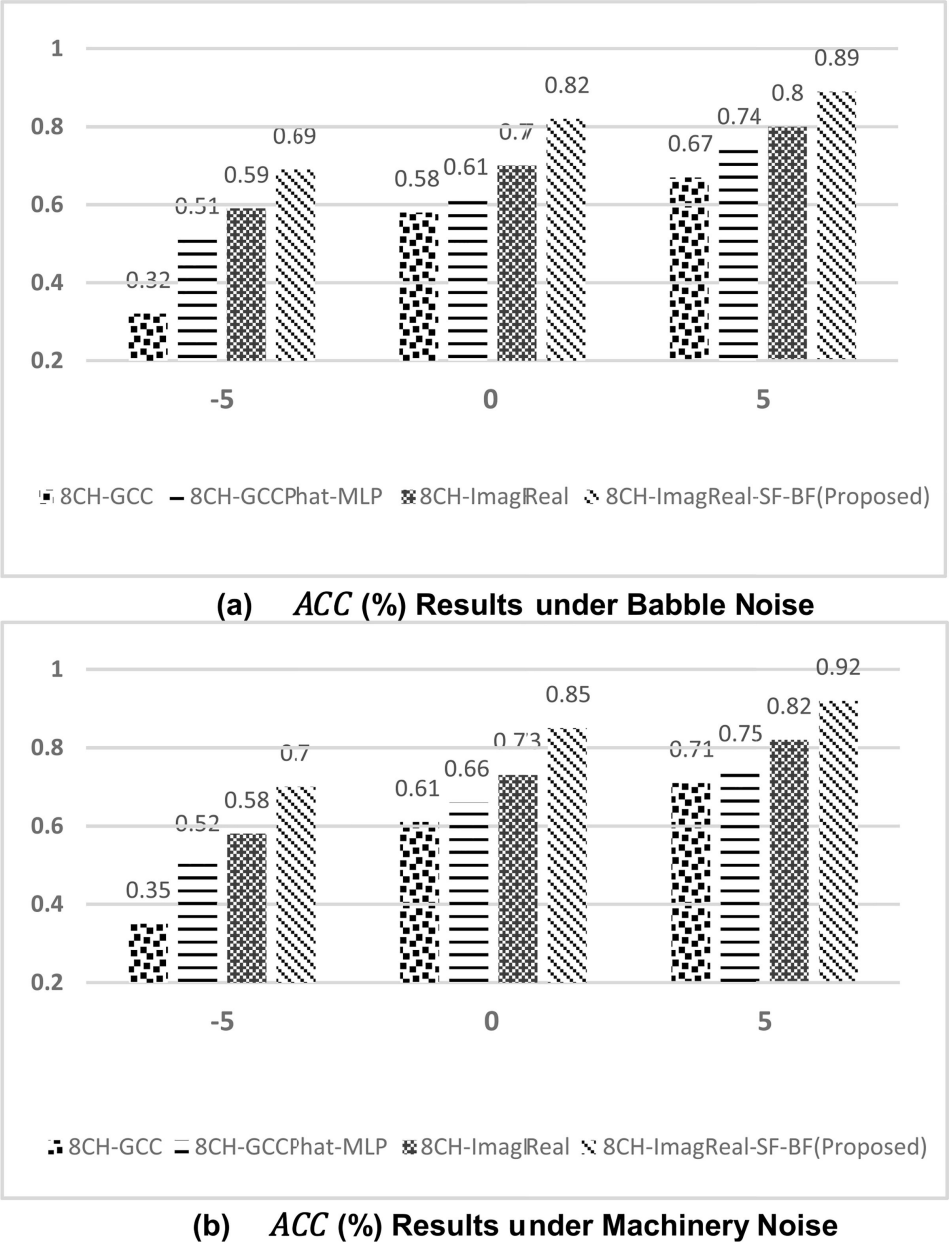
The offline *ACC* results (%) under (a) babble (b) machinery noise conditions. −5, 0 and 5 represent different SNR (in dB) conditions. Both of the training and testing used the collected data in Room A, B, C.

**FIGURE 10.**
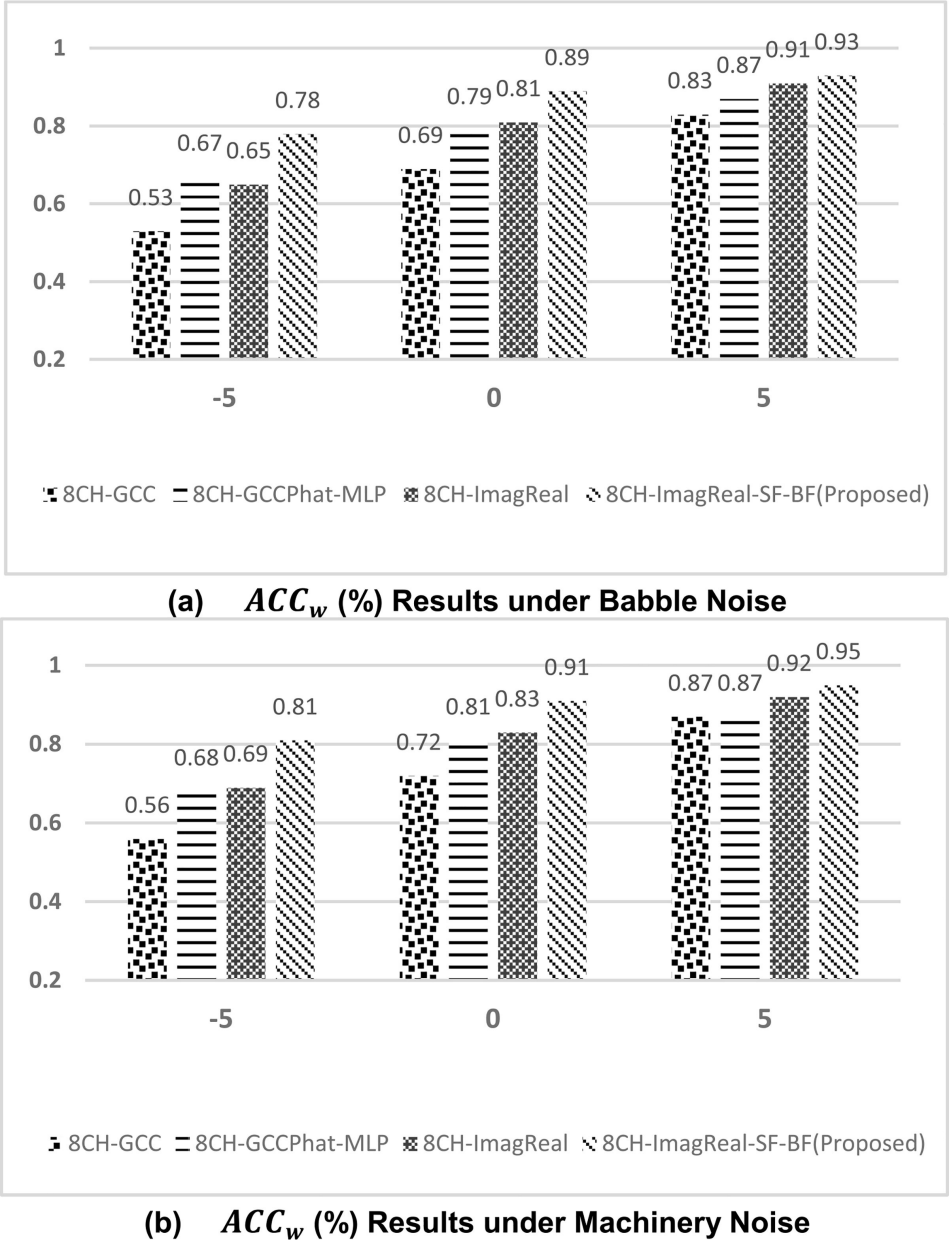
The offline *ACC*_*w*_ results (%) under (a)babble (b)machinery noise conditions. −5, 0 and 5 represent different SNR (in dB) conditions. Both of the training and testing used the collected data in Room A, B, C.

To additionally test the performance of the proposed method, 90-minutes collected noisy data under different noise conditions are used. Fig. 8 presents the confusion matrix of the proposed method when training with the babble noise and machinery noise at 5dB SNR. Both of the performances are superior under two different types of noises. Fig. 9 also shows the offline *ACC* results under machinery and babble noise, and it covers three different SNR levels. To compare the proposed method to other estimators, another three CNN-based estimators are measured. 8CH-GCC is included as a conventional signal-processing based estimator. 8CH-GCCPhat-MLP and 8CH-ImagReal are also included because they are the best two published estimators besides the proposed method in the previous measurement. The offline *ACC* results show that the proposed method is robust to background noise even in low SNRs under babble noise (as one of the toughest noisy situations – a non-stationary noise). The *ACC* of the proposed method at 0dB SNR under machinery noise is above 85%, and even reaches 92% when the SNR is enhanced to 5dB. Fig. 10(a) and (b) show the *ACC*_*w*_ results of the proposed method and comparisons. Under machinery noise, the proposed method gets 95% *ACC*_*w*_ at 5dB SNR, and still gets 81% *ACC*_*w*_ at −5dB SNR. Under babble noise, the *ACC*_*w*_ of the proposed method is slightly lower than the *ACC*_*w*_ under machinery noise, but still more robust to background noise than other comparisons.

## VI. REAL-TIME IMPLEMENTATION AND REAL-TIME MEASURED RESULTS

**FIGURE 11.**
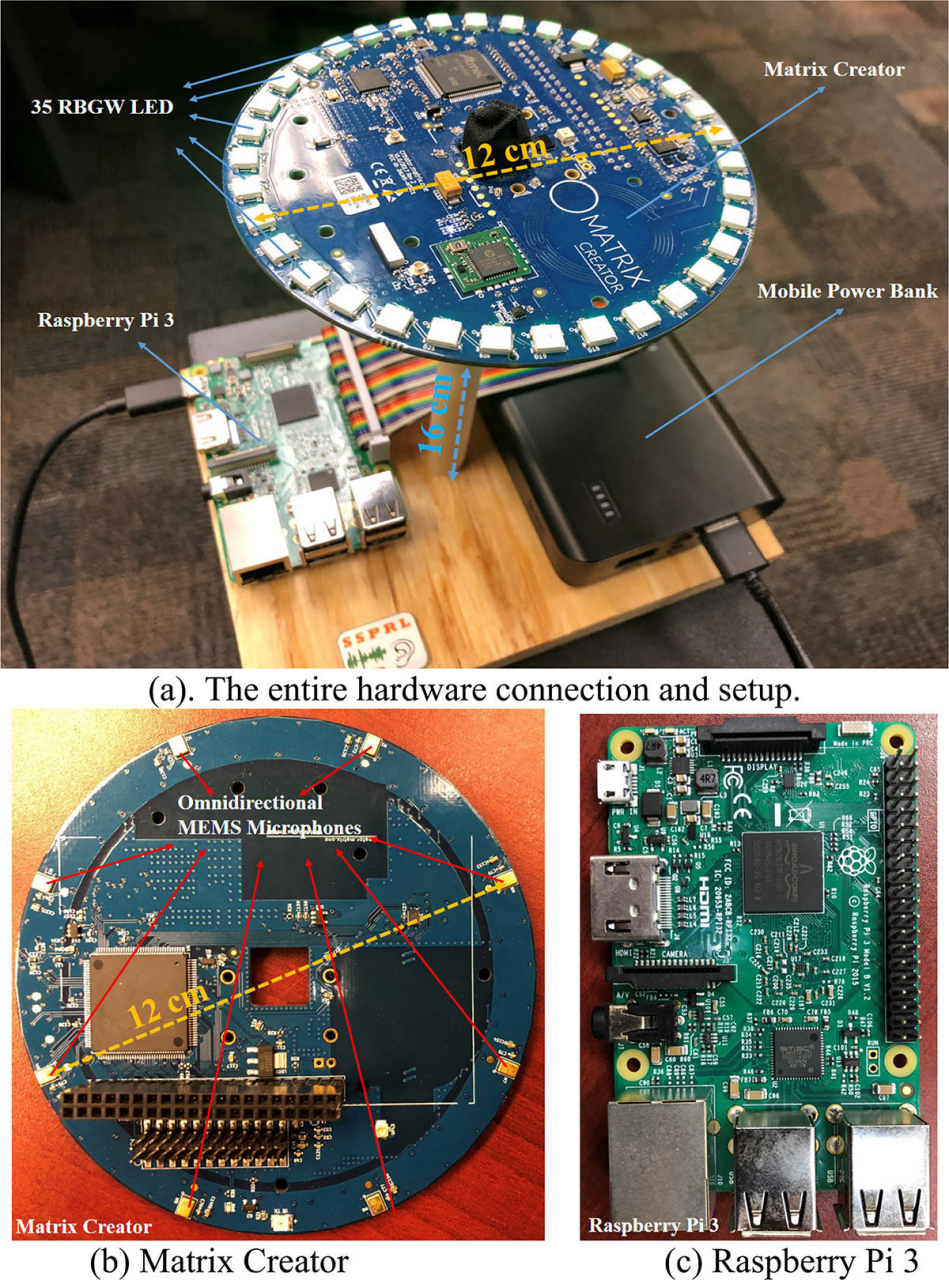
Hardware of the real-time implementation (a)The entire hardware connection and setup(b)Matrix Creator(c)Raspberry Pi 3.

Offline results can partially prove and show the performance of the methods. However, it is always necessary to implement the method in real-time, capture the realistic data, and test it on the fly. The proposed method and several other comparisons have been implemented in real-time. The algorithms are written in C/C++ and Python-based on frame-based data. A single-board computer - the Raspberry Pi 3 (RP3) [31], and an IoT development board - matrix creator (MC) [32] have been used as the real-time implementation platform. Such platform has been used as the recording device as well in the proposed data collection sessions. Fig. 11. shows the hardware platform for real-time implementation. The RP3 and a mobile power bank are sitting on the bottom. The MC with the microphone array is lifted sixteen centimeters high in order to reduce the sound reverberation and reflection effects from the table.

### A. HARDWARE PLATFORM

**FIGURE 12.**
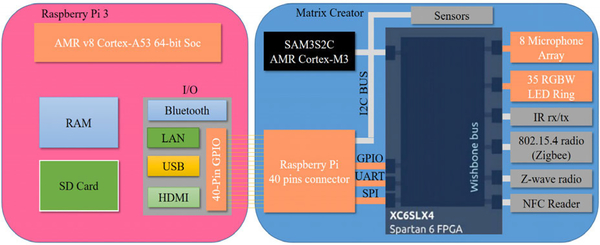
The details of the hardware of the prototyped platform.

As we discussed above, two hardware modules have been used as our hardware platform for real-time implementation. The first one is a single-computer RP3, and another one is an IoT development board of MC which is an extendable board for RP3 via the 40 pins general-purpose input/output (GPIO) connection. In MC, eight-microphone UCA (omnidirectional MEMS microphones) is located at the edge of a small round board on the backside. 35 RBGW-LED lights are also located at the edge of the board as a ring covering 360° on the front side, see Fig. 11(b). Both microphones and lights are controlled by a Spartan 6 FPGA board. The details of the hardware of the prototyped platform is shown in Fig. 12.

### B. FRAME-BASED ALGORITHM IN C/C++ AND PYTHON

**FIGURE 13.**
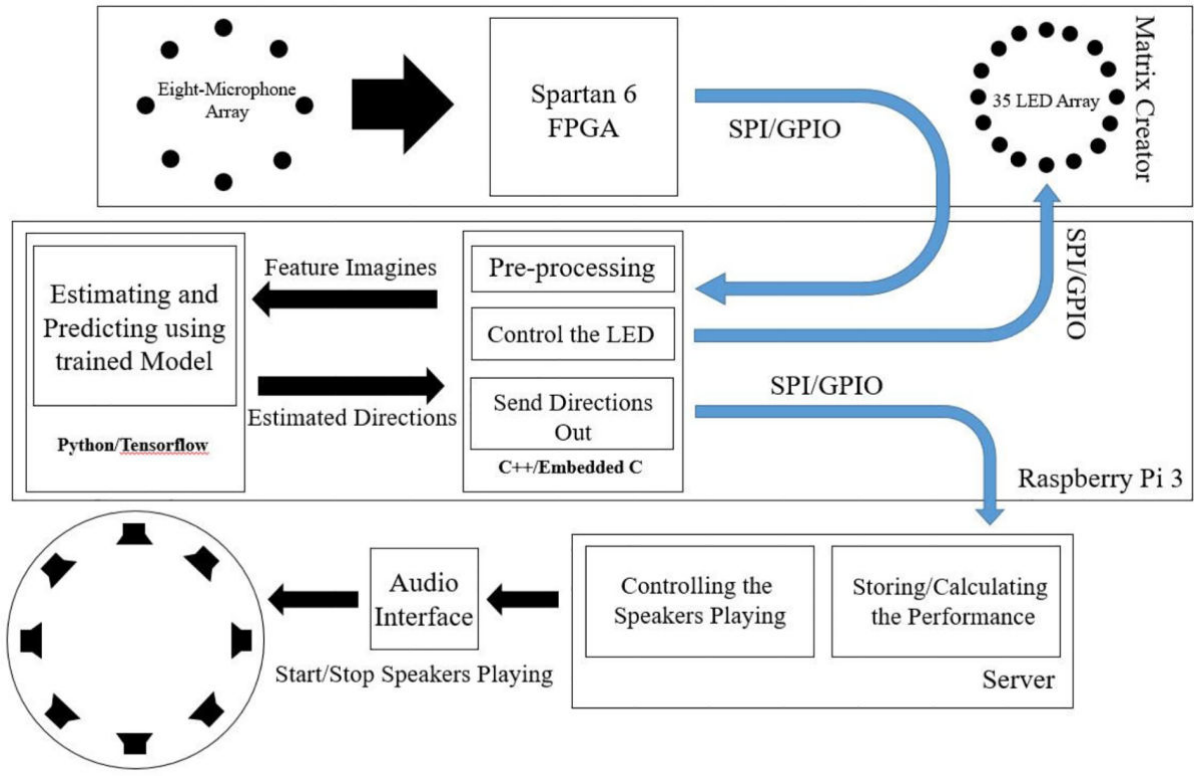
The block diagram of the real-time implementation.

In order to implement the proposed method in real-time, the pre-trained model is frozen. The proposed CNN model is put into the RP3 running in Python on a Linux operating system (OS) using Tensorflow. The computations need to be reduced so that the RP3 is sufficient to handle the real-time processing. The block diagram of the real-time implementation is presented in Fig. 13. The speech signals are captured via the eight-microphone array from the MC board. The microphones on MC are all digital MEMS, which means the output signals have already been converted to digital data from analog. Spartan 6 FPGA gathers and buffers the signal data, then it directly sends them into the RP3 via a serial port protocol - the serial peripheral interface (SPI). In the RP3, an executable file takes control to receive the speech signal data from SPI. The executable file is written in C++ and embedded C and then compiled by GNU [33] compiler collection. In the executable file, the received speech signals are pre-processed to generate the feature maps. Then the feature maps are sent to the pre-trained frozen model, and the model will estimate and predict the direction (DOA angle) based on the input feature maps. Once the estimated direction angle ${\hat{\theta }}_{k}$ is produced, the executable file will then light up the corresponding LED in the MC surface (via SPI) to display the estimated direction of the speech source. Furthermore, in order to evaluate the real-time performance, the estimated direction was sent to the server as well via SPI. The server controls the loudspeakers playing, meanwhile calculating the real-time estimation results. The video clips of the prototype running in real-time are presented in [34].

### C. REAL-TIME PERFORMANCE OF THE PROPOSED METHOD UNDER NOISY CONDITIONS

**FIGURE 14.**
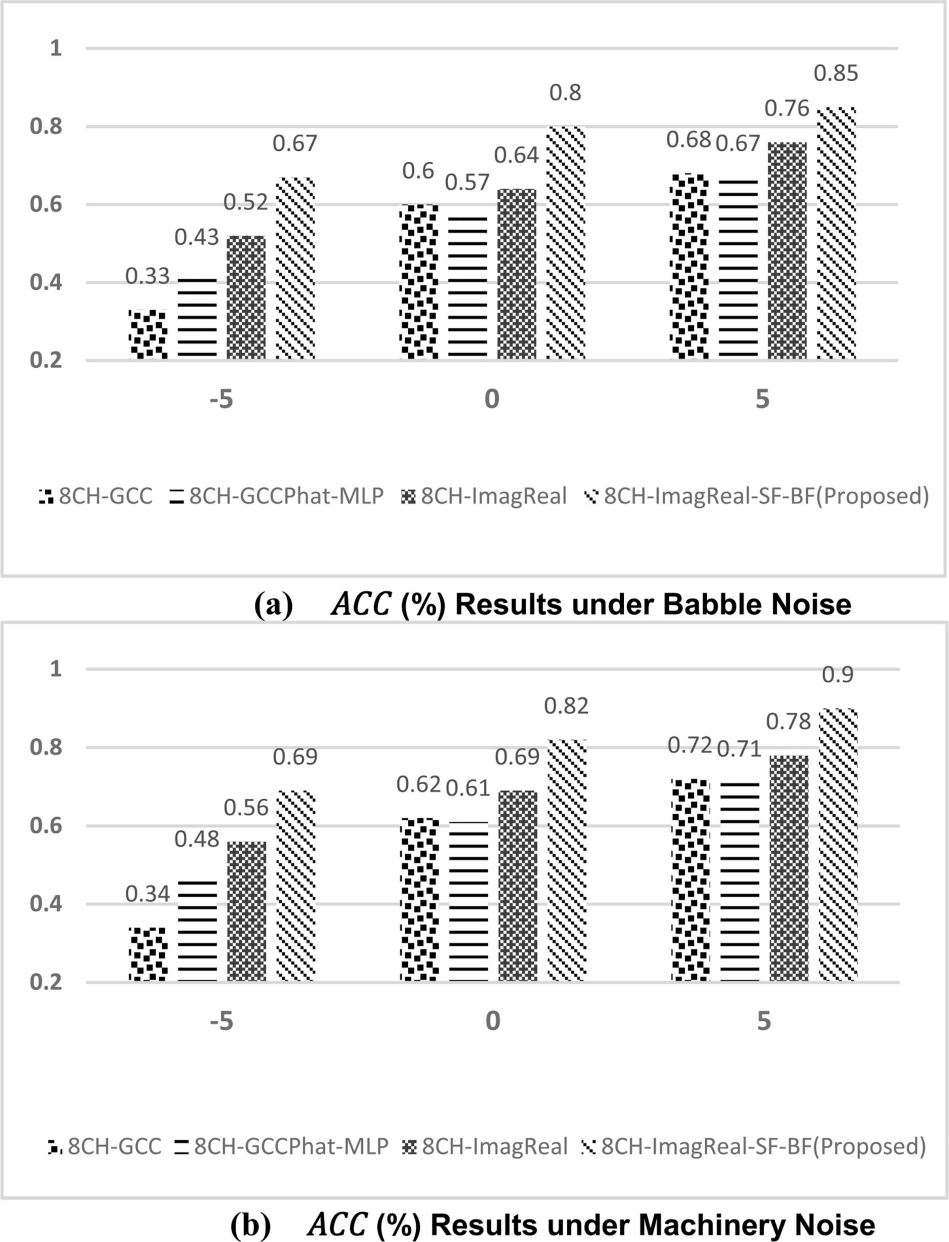
The Real-Time *ACC* results (%) under (a)babble noise (b)machinery noise conditions. −5, 0 and 5 represent different SNR (in dB) conditions. The tests were completed at Room D.

**FIGURE 15.**
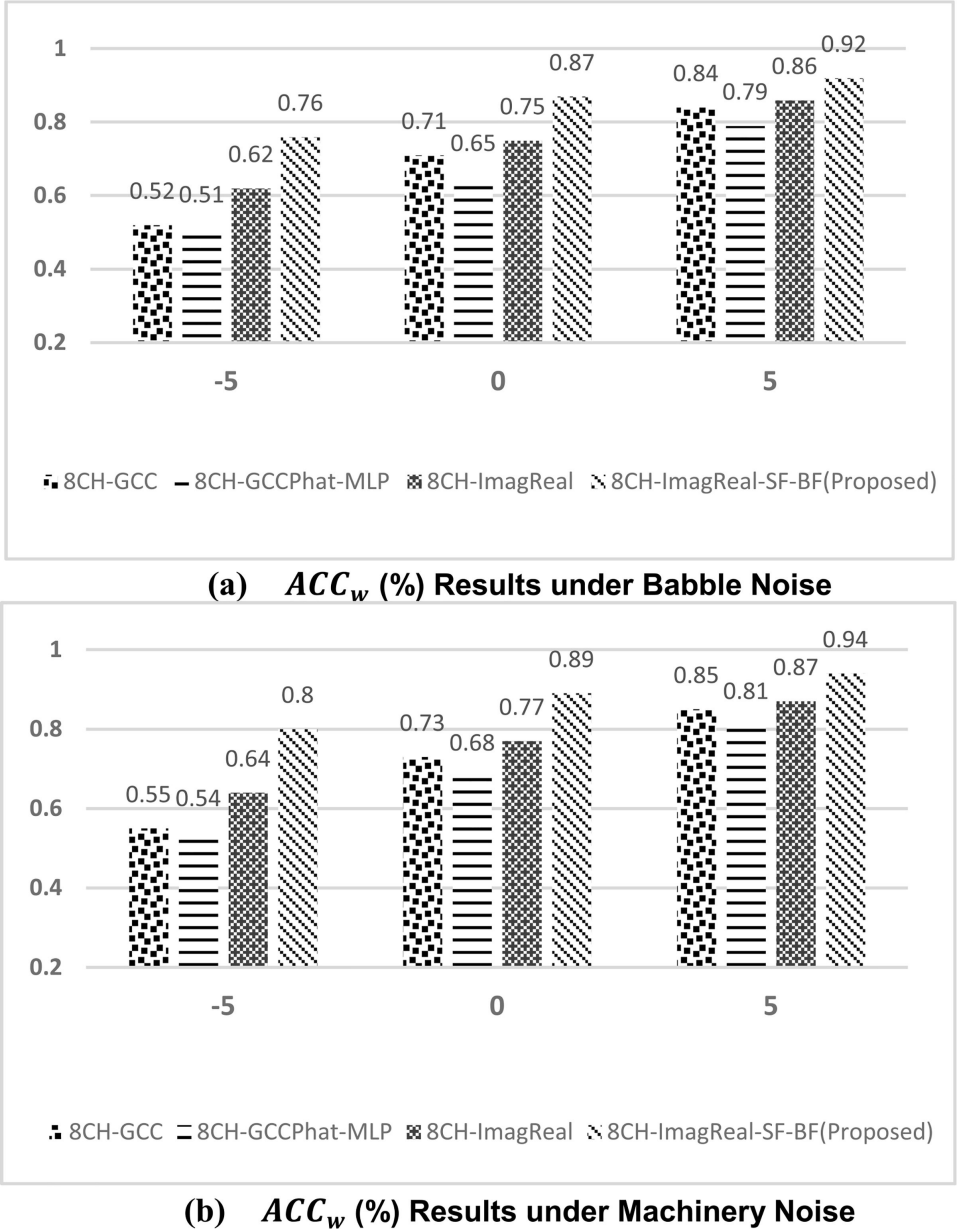
The Real-Time *ACC*_*w*_ results (%) under (a)babble noise (b)machinery noise conditions. −5, 0 and 5 represent different SNR (in dB) conditions. The tests were completed at Room D.

The real-time performance of the proposed method was tested via the prototyped platform. The comparisons including 8CH-GCC, 8CH-GCCPhat-MLP and 8CH-ImagReal are implemented on the same platform as well. The experiments were completed in room which is different as to the data collection rooms. The experiments were under babble and machinery noise with 90-minute speech played from the loudspeakers. The *ACC* and *ACC*_*w*_ results are presented in Fig. 14 and Fig. 15. In our experiments, both of the *ACC* and *ACC*_*w*_ results of 8CH-GCCPhat-MLP are decreased extremely comparing to the offline test. The reasons include (i) the real-time processing may introduce interference and calculation delay to jeopardize the performance, (ii) the model of the 8CH-GCCPhat-MLP is overfitted to the training data. Although the real-time performance of all estimators is degraded (compared to offline performance), the proposed estimator still reaches the best results with *ACC* and *ACC*_*w*_, even when the SNR is low (equal or lower than 0dB). Such real-time measured results show that (i) the proposed method is not overfitted to the training data, (ii) the proposed method is more robust to background noise over the comparisons. The proposed method can be furtherly built as a final/commercial HI product by including other processing modules such as a VAD detector, an auditory processing module, or a speech enhancement module.

### D. THE POWER CONSUMPTION OF THE PROTOTYPE PLATFORM

**FIGURE 16.**
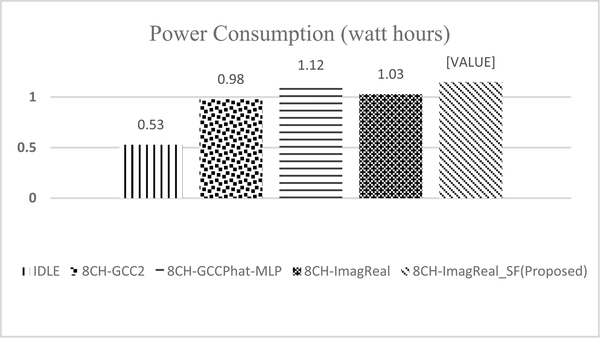
Power consumption of the prototype (watt hours).

To develop a robust SSL estimator in real-time, the power consumption is therefore important to consider. In our hardware setup, the capacity of the power bank sitting on the bottom (Fig. 11(a)) is 20k milliamps per hour. Our power consumption measurement has been completed with the fully charged power bank, the results are presented in Fig. 16, where Y-axis shows the watts consumption per hour, and X-axis shows the methods. In Fig. 16, “IDLE” stands for the power consumption of the prototype operating system running without any extra processing or calculation. The total power consumption of the platform for the proposed method, including all processing stages, is only 1.15 watts per hour, comparable to the power consumption of 8CH-GCCPhat-MLP. Since our setup is only a prototype unit using the development boards, the power consumption shown here is much more than what is needed for the implementation of the proposed method. This is so since many other unnecessary modules unrelated to the proposed method are also running on the boards. The end-product, as a dedicated hearing improvement unit, will only need to keep and run the modules required for the implementation of the proposed method, hence the power consumption will be very small. Additionally, the size of the end-product will be much smaller and compact compared to the prototype platform.

## VII. CONCLUSION

In this article, we proposed a CNN-based SSL estimator using an eight-microphone UCA. Imaginary-real coefficients and spectral flux are used as feature set for the CNN model. Beamforming is used as well to enhance the SNR when computing the spectral flux. The offline and real-time results show that the proposed SSL method, as an augmentation method for imaginary-real coefficients CNN based DOA method, is scalable and robust under different types of noise and performs better than other neural network based estimators. A prototype platform for implementing the proposed method in real-time was also developed using a single-board computer, Raspberry Pi, plus an IoT development board. The prototype platform not only shows the robustness but also presents and establishes a real-time platform. The end-products including HI devices can be built based on the platform with a VAD (to “freeze the estimation” when no speech detected). Such products help to improve the hearing capability of people with hearing loss by identifying the direction and location of the speakers in noisy environments and where there maybe several people such as in a group meeting or a social gathering.

## Figures and Tables

**TABLE 1.** Collection setup.

	Quantity	Details
*Room A*	1	7 × 4 × 2.5 m (RT60: 0.4s)
*Room B*	1	4 × 4 × 3 m (RT60: 0.3s)
*Room C*	1	8 × 4 × 3 m (RT60: 0.4s)
*Room D*	1	6 × 4 × 3 m (RT60: 0.3s)
*Speech Loudspeaker*	8	Fostex 6301B
*Noise Loudspeaker*	1	Bose SoundLink Mini II
*Circular Table*	1	1.2 meters diameter
*Audio Interface*	1	Focusrite Scarlett 18i20
*Recording Device*	1	Matrix Creator (8 MEMS MIC)
*Clean Speech*	90 min	HINT Database
*Noise*	2 Types	Babble & Machinery
